# Variations in plantar pressure and balance in HIV-infected children in antiretroviral therapy

**DOI:** 10.1038/s41598-019-41028-0

**Published:** 2019-03-13

**Authors:** Lucieny da Silva Pontes, Bianca Callegari, Lizandra Magno, Anderson Moraes, Bruno Giovanni Silva, Kaio Manso, Brenison Barros, Ana Paula Araújo, Maria Clara Silva, George Alberto Dias, Beatriz Helena Vasconcelos, Anselmo Costa e Silva, Rosana Maria Libonati, Givago Silva Souza

**Affiliations:** 1 Universidade do Estado do Pará, Belém, Pará Brazil; 2 Universidade Federal do Pará, Belém, Pará Brazil

## Abstract

Balance disorders have been poorly investigated and somewhat neglected in people infected with the human immunodeficiency virus, especially in children, whose have intrinsic and extrinsic risk factors that may compromise the balance. To evaluate the foot plantar pressures and the balance in children with acquired immunodeficiency. We recruited 53 children aged between 6 and 15 years: 33 healthy children, and 20 children with positive serology for the human immunodeficiency virus. A physical examination included anthropometric, reflexes, tactile sensitivity of the foot and orthopedic evaluation. We also collected data of them using Pediatric Equilibrium Scale, baropodometry, and stabilometry. We considered significance level of 0.05 for statistics. Both groups were aged-, sex-matched and similar body mass index and scores of the Pediatric Equilibrium Scale. Three infected children had altered tactile sensitivity, and none had orthopedic or reflex alteration. Infected children had higher mean plantar pressure in the hindfoot than of the control group (p = 0.02). There was higher maximum plantar pressure in the hindfoot of the infected children than of the controls (p = 0.04). Controls had lower maximum plantar pressure in the forefoot than the infected children (p = 0.04). Infected children had larger displacement of the center of pressure (p = 0.006), larger mean velocity of displacement (p = 0.006), and longer duration between successive peaks of displacement than the controls (p = 0.02). Children living with the human immunodeficiency virus discharges great plantar pressures in the hindfoot and to present balance disturbances in the absence of neurological symptomatology.

## Introduction

Despite the advances in the prophylaxis and treatment of the human immunodeficiency virus (HIV) infection, it still impacts a large number of individuals^1^. About 37 million people in the world is infected with HIV, along with 1.8 million children under 15 years^2–4^. The transmission of HIV from the HIV-infected mother to her child during pregnancy is concerning because the virus infection will occur in the first ages of the neuropsychomotor development^5^.

Previous investigations have reported deficits in cognitive, sensory, and motor functions in HIV-infected individuals^6–8^. These deficits compromise psychomotor abilities, visuomotor integration, visuospatial perception, and language^7^. Clinical evidence of balance and gait disturbances were frequently detected in HIV-infected adults^9–12^. These investigations reported distal neuropathy that would impair balance and vibrotactile sensation. One possible consequence of the deficits in the postural control is the load distribution on the plantar surface of the foot^9^. The topographical recording of pressures on the plantar surface of the foot could indicate imbalance and overload on the foot’s structure^13,14^. Balance disorders have been poorly investigated and somewhat neglected in HIV-infected people.

The exposure to Antiretroviral Therapy (ART), tropism of the HIV to the nervous system, and the children nervous system development can turn this HIV-infected population more susceptible to deficits in postural control and its functional repercussions. The present investigation compared a quantitative evaluation of balance and plantar foot pressure of HIV-infected and non-infected children.

## Results

### General features

No patients had deformities and spasms in the limbs, as well as they also have normal reflexes. The areas of the feet of the subjects were identified as altered sensitivity from the perception with the violet filament, considering 9 areas in the plantar surface. HIV+ subjects 4, 5, 6, 7, 8, 9, 12, 14, 17, 18 and 19 were considered with altered sensitivity. When analyzing the proprioceptive sensitivity through the diapason, no alteration was found for this type of sensitivity in both groups.

Table 1 shows the general information of the sample from each group. Both groups were age- and sex-matched, and they had similar physical features (weight, height, and body mass index). Both groups also had non-significant differences for the pediatric balance scale.

**Table 1:** Comparison of the general features of control group and HIV infected group (HIV+).

Variables	Groups		*p-value*
	Control	HIV+	
*Sex (F/M)*	12 F/21 M	8 F/12 M	0.97
*Age (years)*	9.5 ± 2.4	9.6 ± 2.6	0.99
*Weight (Kg)*	36.55 ± 15.77	29.16 ± 8.9	0.12
*Height (cm)*	131.7 ± 33.8	130.7 ± 13.8	0.15
*BMI*	18.2 ± 4.2	16.6 ± 2.3	0.31
*PBS*	55.3 ± 0.9	55.1 ± 1.2	0.51

F: female; M: male; BMI: body mass index; PBS: pediatric balance scale.

Table 2 shows the individual features for viral load, CD4 and CD8 counting, duration of the ART and the drug therapy they have.

**Table 2:** Laboratorial characterization of the HIV+.

Subjects	Viral load (copies/mm)	CD4 (cells/mm)	CD8 (cells/mm)	ART duration (years)	Drug therapy
*S1*	1.558	579	1.300	9	ddl/3TC/LPV/r
*S2*	<40	752	796	6	EFZ/3TC/AZT
*S3*	559.651	21	1.307	9	LPV/r/AZT/3TC
*S4*	283.52	388	3.041	11	EFZ/AZT/3TC
*S5*	1.315	486	603	10	EFZ/AZT/3TC
*S6*	4.326	816	2.785	4	NVP/3TC/AZT
*S7*	ND	202	1.598	6	EFZ/AZT/3TC
*S8*	147	564	1.479	5	EFZ/AZT/3TC
*S9*	36.435	1.364	899	6	LPV/3TC/AZT
*S10*	<40	564	1.479	5	LPV/3TC/AZT
*S11*	ND	1.364	899	6	EFZ/AZT/3TC
*S12*	124	628	674	9	LPV/3TC/AZT
*S13*	63.328	431	699	5	EFZ/3TC/AZT
*S14*	ND	1.281	1.117	7	EFZ/AZT/3TC
*S15*	ND	712	507	7	EFZ/AZT/3TC
*S16*	NI	NI	NI	2	AZT/3TC/NVP
*S17*	NI	NI	NI	5	AZT/3TC/NVP
*S18*	521.149	146	1.115	10	EFZ/AZT/LPV
*S19*	75	965	936	8	AZT/3TC/KALETRA
*S20*	2.346	1.519	1.866	2	AZT/3TC/NVP

ART: antiretroviral therapy; ND: non-detectable; NI: non-informed; ddl: Didanosine; 3TC: Lamivudina; LPV/r: Lopinavir + ritonavir; EFZ: Efavirez; AZT: Zidovudina; NVP: Neviparina.

### Plantar foot surface pressure

Tables 3 and 4 compare the results of the medium and maximum plantar pressure between both groups, respectively. Both groups had an expected load distribution on the feet featuring a higher load on the hindfoot compared to the load on the midfoot and forefoot. The comparison of the mean plantar pressure between both groups for the open-eyed condition had no significant difference in any area of the foot. In the hindfoot, both areas (MH and LH) were in the border of statistical significance (p = 0.05). For the CE condition, the comparison of the mean plantar pressure also had no significant intergroup difference except in the area LH of the hindfoot. The participants of the HIV+ had higher plantar pressure in the hindfoot than the controls (p < 0.05, statistical power = 60%).

**Table 3:** Comparison of the mean plantar pressure, in kPa, (10^−3^ × mean ± standard deviation) between control group and HIV+.

Region	Open-eyed condition			Closed eye condition		
	Control	HIV+	*p-value*	Control	HIV+	*p-value*
*T1*	32.8 ± 26	23.8 ± 36.5	0.3	38 ± 30.8	50.5 ± 48.7	0.67
*T2-5*	12.6 ± 14.7	7.4 ± 16.7	0.25	12 ± 15.8	4.9 ± 9.6	0.1
*M1*	94.4 ± 21.7	98.3 ± 17.3	0.51	98.7 ± 19.5	100.1 ± 19.4	0.8
*M2*	112.5 ± 20.1	106.4 ± 21.8	0.3	111.7 ± 20.6	107.7 ± 22.1	0.62
*M3*	115 ± 23.4	108.4 ± 23.7	0.33	113.8 ± 22.7	112.5 ± 21.5	0.83
*M4*	113.6 ± 24.5	105.3 ± 18.3	0.2	112.8 ± 25.1	103.9 ± 20.6	0.19
*M5*	89.9 ± 18.2	87 ± 13	0.54	87.4 ± 18.7	87.9 ± 14.3	0.91
*MF*	69.9 ± 26.1	64.3 ± 34.8	0.51	69.8 ± 27.7	64.9 ± 36.2	0.58
*MH*	188.3 ± 34.2	209 ± 40.9	0.05	188.5 ± 37.8	207.4 ± 35.5	0.08
*LH*	171 ± 30.9	190.1 ± 38.5	0.05	**167.3** ± **34.5**	**190.4** ± **36.2**	**0.02**

The comparisons with significant differences are in bold.

**Table 4:** Comparison of the maximum plantar pressure, in kPa, (10^−3^ × mean ± standard deviation) between control group and HIV+.

Region	Open-eyed condition			Closed eye condition		
	Control	HIV+	*p-value*	Control	HIV+	*p-value*
*T1*	33.6 ± 31.4	21.1 ± 41	0.22	**38** ± **32.2**	**18.8** ± **31.6**	**0.04**
*T2-5*	12.5 ± 16	7.7 ± 18.6	0.33	**12.7** ± **14.9**	**2.8** ± **5**	**0.02**
*M1*	95.7 ± 24.8	92.4 ± 27	0.65	96.5 ± 23.4	94.3 ± 27.9	0.76
*M2*	96.2 ± 18.7	88.1 ± 24.8	0.18	93 ± 19.1	90.2 ± 21.1	0.62
*M3*	93.5 ± 21.1	86.1 ± 22.1	0.22	92.1 ± 21.5	90.7 ± 20.7	0.82
*M4*	92.8 ± 21.3	85.5 ± 18.9	0.21	90.9 ± 23.1	86 ± 22	0.44
*M5*	86.8 ± 22.7	76.5 ± 12.9	0.11	86.5 ± 21.9	78.1 ± 13	0.1
*MF*	73.3 ± 29.4	63.2 ± 35.7	0.27	70.2 ± 33.1	62.4 ± 37.9	0.43
*MH*	214.1 ± 50.1	241.4 ± 61	0.08	213 ± 53.8	240.2 ± 54.6	0.08
*LH*	**201.6** ± **48.7**	**232.8** ± **59.2**	**0.04**	207.2 ± 53.5	236.6 ± 54.8	0.06

The comparisons with significant differences are in bold.

Both groups also had the same profile of the maximum plantar pressure distribution on the foot. The higher loads were found in the hindfoot compared to the other regions of the foot. For the intergroup comparison of the plantar pressures in the EO condition, we observed that again, the HIV+ had a significantly higher load than the control group in the LH area of the hindfoot (p < 0.05, statistical power = 50%). In the other areas of the foot, no significant difference was found. In the CE condition, we observed that there were significant differences in the areas, T1 and T2-5, in which the HIV+ had lower load than the controls (p < 0.05, statistical power = 60–80%). No other areas had significant differences in the intergroup comparison of the maximum plantar pressure.

### Stabilometry

The balance evaluation results are shown in Table 5. We observed that HIV+ had larger COP displacement and higher speed than the controls for both EO and CE conditions (p < 0.05, statistical power = 80–85%). We also observed that HIV+ had less MT values than the controls in the opened-eyed condition (p < 0.05, statistical power = 60%). No other comparison had significant intergroup difference.

**Table 5:** Comparisons of the posturographic results between the control group and HIV+.

Global variables	Open-eyed condition			Closed eye condition		
	Control	HIV+	*p-value*	Control	HIV+	*p-value*
*X axis*	2.7 ± 1.5	2.45 ± 0.9	0.81	3.2 ± 2.1	2.42 ± 1	0.32
*Y axis*	3.6 ± 1.9	3.4 ± 1.5	0.67	4.35 ± 2.1	3.44 ± 1.4	0.1
*CoP disp*.	**923.7** ± **638.1**	**1545.3** ± **899.4**	0.004*	**993.7** ± **621.9**	**1579.44** ± **845.6**	0.006*
*Speed*	**15.4** ± **10.6**	**25.8** ± **15**	0.005*	**16.6** ± **10.3**	**26.3** ± **14.1**	0.006*
*Area*	268.7 ± 370.1	208.9 ± 166.3	0.63	332.8 ± 358.2	200.5 ± 156.9	0.23
*MP*	7.36 ± 5.2	5.62 ± 3.9	0.2	5.3 ± 3.8	4.1 ± 2.9	0.26
*MD*	2.62 ± 2.2	2.44 ± 1.1	0.35	3 ± 1.91	2.7 ± 1.19	0.87
*MT*	**2.36** ± **0.5**	**2.69** ± **0.5**	0.02*	2.47 ± 0.57	2.74 ± 0.57	0.1
**Romberg quotient**						
**Variables**	**Control**		**HIV+**		***p-value***	
*X axis*	1.01 ± 0.5		1.12 ± 0.5		0.44	
*Y axis*	0.88 ± 0.4		1.05 ± 0.4		0.13	
*CoP disp*.	0.96 ± 0.4		0.98 ± 0.2		0.16	
*Speed*	0.95 ± 0.3		0.98 ± 0.2		0.14	
*Area*	1.15 ± 1.9		1.22 ± 0.7		0.09	

*CoP disp*: displacement of the centre of pressure; *MP*: mean peak value in the Sway density curve; *MD*: the mean distance between successive peaks in mm; *MT*: mean time interval between successive peaks. The comparisons with significant differences are in bold.

## Discussion

This study aimed to verify the impact of HIV infection and the use of antiretroviral therapy on the pressure plantar and static balance of children and adolescents living with AIDS. The study identified a tendency of weight load in the region on the hindfoot in HIV+ participants and change in the postural control indicated by the variables CoP distance, mean velocity, and MT.

The foot is the only ground attachment during bipedal locomotion. It has an important function in supporting body load, impact dampening, and the maintenance of postural stability, and it adapts to the structural and functional changes that occur throughout life^15–18^. A developing child can stand independently at 12 months of age, and after this motor acquisition, there is a specific and continuous refinement of postural control throughout childhood^19^.

Previously, other investigations have shown posturographic changes in asymptomatic and symptomatic adult patients infected with HIV (see a review in Berner *et al*.)^20^. The studies have investigated the balance disturbances using different methods such as measurements of centre of pressure oscilation, recordings of postural reflex latency, and timed clinical test. Trendwalker *et al*.^12^ observed that HIV-infected patients had postural instability, especially when they were in unstable foot support under a force platform. Arendt *et al*.^10^ used a force plate to study 36 HIV-infected subjects, but asymptomatic for neurological dysfunctions, and 10 HIV-infected subjects in the early stage of encephalopathy. They observed that either the patients with encephalopathy either 25% of asymptomatic individuals had disturbance in the postural control. Delepiane *et al*.^11^ recorded static and dynamic posturagraphic using force platform also demonstrated individuals with advanced HIV/AIDS, as well as HIV-infected patients with asymptomatic neurology, exhibited altered postural control when compared to a control group. Bauer *et al*.^9^ carried out a study on 28 HIV-infected subjects without ART, 25 HIV-infected subjects using nucleoside analog inhibitors, 37 HIV-infected subjects using ART, and 78 subjects without positive HIV serology. They identified a decrease in postural control and abnormalities in the vibrotactile threshold in the three HIV-infected groups during the test on the platform with eyes closed^9^. The vibrotactile threshold was influenced by the type of ART, while the balance was not. However, these alterations, even when present, were considered to be moderate in relation to the control group. Our results showed that similar results observed in a HIV-infected adult sample were also found in a HIV-infected children sample.

Neurological manifestations in individuals with HIV/AIDS are well documented in the literature^6–8,21^. For Christo^8^, the most important determinant of vulnerability to structural damage to the nervous system is the degree of immunosuppression. Neurological manifestations compromise about 40% to 70% of HIV carriers during the course of the infection, and in post-autopsy studies, these values approach 90% of the cases. Blanchette *et al*.^7^ reported important abnormalities in HIV-infected children (impairment in the process of myelination and the presence of subcortical lesions) were found in imaging tests (computed tomography) and were associated with delayed motor and cognitive development. The involvement of the nervous system during the course of HIV infection is notorious. However, the frequency and timing of clinical manifestations, as well as the interactions with other risk factors for the development of developmental delays (and, consequently, deficiencies), are still not well characterized^6–8,22^. Our HIV-infected sample had no deformities and normal reflexes. Few of them had some tactile sensitivity loss, but in general, the HIV-infected children were neurologically asymptomatic, and they had normal balance when evaluated by PBS. Our results indicated that the stabilometry and baropodometry could be used to detect subclinical neurological losses in HIV-infected children and enable us to evaluate the neurological status of HIV-infected children.

The children we studied presented at least two conditions of vulnerability of nervous system involvement, either by the natural course of the disease as either by prolonged exposure to ART. In this study, postural control in HIV+ children had altered results for the variables COP distance, mean velocity, and MT parameters. These three findings are indicators of the posture instabilibity of the patients. The increase of the COP distance of the HIV+ indicated greater oscilations than the controls. The time between successive peaks (MT) was also longer for the HIV+ with faster mean velocity than the controls.

Central postural control depends on the entrance of three peripheral modalities (vision, vestibular apparatus, and proprioception)^23,24^. The somatosensory, visual, and vestibular systems must be integrated to recognize and interpret complex sensory environments and act in conjunction with the motor and cognitive systems to provide inputs to maintain body stability^25^. In this study, the visual assessment was suppressed in the EC condition, but the surface of contact of the feet and the scenario were not modified—that is, there were no disturbances generating instability for the proprioceptive and vestibular systems, respectively. The absence of alterations in the Romberg index for the different parameters allowed to exclude the visual impairment from the balance loss^26^, that probably reflect loss in proprioceptive or vestibular functions, which need to be further investigated.

The baropodometric results we obtained indicated a tendency of the HIV-children to displace the body load in the hindfoot more than the controls. We found that infected children had higher load in the LH area of the hindfoot, and lower load in the T1, and T2-5 areas than controls. We found no differences in the BMI or even in the body structure. Initial stages of gait, there is greater pressure on the hallux, with progressive distribution to almost all the regions of the foot with the increase of the age, followed by a decrease of the pressures on the midfoot, consequently to the appearance of the arches plantar and with progression growing and continuous to the back, around 8 years of age until adulthood^15,16^.

Studies in populations with infectious diseases have been anticipating subclinical balance abnormalities in infected but still asymptomatic patients. Vasconcelos *et al*.^13^ compared the distribution of body weight in different areas of the feet in patients with human T-cell lymphoma virus type 1 (HTLV-1) associated with myelopathy/tropical spastic paraparesis (HAM/TSP) and inferred that asymptomatic patients with HTLV-1 presented higher plantar pressures in the forefoot region as well as those with neurological manifestations and with HAM/TSP with a gait characterized by greater pressure distribution in the forefoot region. These patients presented spasticity of the lower limbs with consequent equinism of the feet, a fact that justifies the greater plantar pressure in the forefoot in symptomatic individuals and, thus, signals possible early involvement of the nervous system in asymptomatic individuals. In this way, they would be better investigated in populations with infectious and chronic diseases in which the etiological agent has a predilection to undermine the nervous system and cause subclinical signs of balance abnormalities since the viral latency stage can mimic possible damage to the nervous system and abnormalities in postural control.

There is an imminent risk of aggression to the nervous and muscular systems, which need to be monitored, so with this study we intend to signal subclinical findings that are poorly investigated and neglected in follow-up services for people living with HIV/AIDS. Our children and adolescents are in a situation of clinical and social vulnerability; the clinical conditions have already been exposed above and, socially, because they live in a unique region in relation to their geography, such as the Amazon region, with difficult access to services and continuity to the treatment, as well as having low educational level and living in poverty. Early detection of postural control abnormalities may lead to the development of assessment protocols, establishment of guidelines in health services, training of professionals for monitoring and treatment by rehabilitation staff, including the physical therapist, to minimize the damages caused by the aggressive effects of HIV and its drug therapies.

In conclusion, according to the progression of age, the plantar pressures distribute to the regions of the forefoot and back, mainly due to the appearance of the plantar arches. HIV+ Children and adolescents presented greater weight load in the hindfoot region, specifically in the LH area in closed eyes, perhaps signaling involvement of sensory pathways. These presented subclinical postural instability, since abnormalities in postural control were detected for the variables CoP distance, mean velocity and mean time between successive peaks of CoP.

## Methods

### Subjects

Fifty-three children aged under 15 years were divided into two groups: a control group (CG) composed of 33 children (21 males, 12 females, 9.5 ± 2.4 years-old) with negative HIV serology, and AIDS group (HIV+) composed of 20 children (12 males, 8 females, 9.6 ± 2.6 years-old) infected vertically by the HIV. HIV-infected individuals had received ART for at least 1 year and were recruited from a Mother and Child Reference Unit of the Belém city, Brazil, reference in treatment and follow-up of HIV-infected children.

We excluded HIV-infected children with active pulmonary opportunistic diseases, congenital heart disease, presence of behavioral alterations, presence of incapacitating physical limitations, gait alterations, vestibular alterations, transmetatarsal amputation, visual deficits, hearing impairment, chronic diabetes, and with hyperhidrosis.

The study was approved by the Ethics Committee of the Federal University of Pará (report #1438). The parent and/or legal guardian of all children were informed about the procedures and have the written consent form agreeing with their participation. In addition, subjects aged 12 or over also signed an informed consent for participation in the survey. All experiments were performed in accordance with the tenets of Helsinki declaration.

### Laboratorial characteristics

It was performed only with the HIV+ for ethical reasons. We obtained, from its more recent clinical recordings, the information of viral load, TCD4 and TCD8 lymphocyte counts, the time of ART initiation, and the drug therapy in use.

### Body Mass Index (BMI)

Anthropometric evaluation was done using a height and weight measuring instrument (Filizola, São Paulo, Brazil) at the moment of the interview. Body Mass Index (BMI) was calculated using Equation 1.

1 $${\rm{BMI}}=\frac{{\rm{W}}}{{{\rm{H}}}^{2}}$$

where BMI is the body mass index, W is the weight in kg, and H is the height in cm.

### Physical assessment

The deformity and muscle spasm evaluation was made by the tests of Thomas and Galeazzi^27^. The evaluation of the patellar and calcaneal reflexes was performed using a reflex hammer, and these were classified as absent, decreased, normal, or increased. The tactile sensitivity of the plantar surface of the foot was investigated by using the Semmes-Weinstein monofilaments, five monofilaments that bent when a force of 0.2, 2, 4, 10, and 300 g was used. The test was first performed with the blue monofilament (0.2 g) that was touched on 9 different areas of the feet (6 forefoot areas, 2 midfoot areas, and 1 hindfoot area), and then the subjects were asked to indicate when and where the touch occurred. Each monofilament was applied three times in the same area. The sensitivity threshold was defined as the lighter monofilament identified by the subject. When the threshold was greater than 2 g, the tactile sensitivity of the skin in this area was considered altered.

### Pediatric Balance Scale

Pediatric Balance Scale (PBS) was developed from the modification of the Berg Balance Scale to obtain a more adequate scale for the children population1^28–30^. PBS is composed of 14 tasks with five items each and scores varying from 0 to 4 for each task: 0 means unable to perform the task and 4 means performs task in an independent way. The total score varies from 0–56 points, with scores between 41 and 56 points being considered low risk of falling, medium risk of falls between 21 and 40 points, and high risk of falls from 0 to 20 points.

### Stabilometric and plantar foot surface pressure measurements

Simultaneous stabilometric and plantar foot pressure measurements were performed using a capacitive pressure platform (EPS/R-1 model, Loran Engineering, Castel Maggiore, Bologna, Italy) with 2224 sensors distributed in 48 cm^2^ and connected to a computer with the Biomech software, with a sample rate of 50 Hz.

The subjects remained with both feet on the platform, one foot apart, with the arm laterally to the body, and 1 m away from a wall with a cross for eye fixation. The tests were performed barefoot with eyes open (EO) and eyes closed (EC). We used a black eye patch to close the eye. The volunteers were asked to maintain a stationary and comfortable posture on the platform and no movement during the 60 seconds of the test. Three trials for both EO and EC conditions were performed, and we analyzed the average of the trials.

For the measurements of plantar pressures, bilaterally, the forefoot, midfoot and hindfoot regions, as well as the specific areas of contact distribution of the feet with the ground were considered: T1 (hallux); T2-5 (other toes); M1, M2, M3, M4, and M5 (heads of each of the metatarsals); MF (midfoot); and MH and LH (medial and lateral hindfoot region). Mean maximum and medium pressure in KPa was analyzed.

For the balance measurements, the following global parameters were evaluated: center of pressure (CoP), CoP velocity, antero-posterior deviation (X-axis), mid-lateral deviation (Y-axis), and area of an ellipse that covers 90% of the total area covered in the mid-lateral and antero-posterior deviations^31–33^. We also estimated structural posturographic parameters from the Sway density curve that were mean peak values (MP) on the Sway density curve, mean time interval (MT), in seconds, between successive peaks, and mean distance (MS) representing the mean distance between successive peaks in mm^33,34^.

For each posturographic parameter, we measured the Romberg quotient, which is the ratio during the evaluation with OE and CE^26^.

### Statistical analysis

Statistical analysis was performed using GraphPad Prism 5.0 software. The D’Agostino-Pearson test was used to evaluate the normality of the data. The variables of the present study had a non-normal distribution, requiring the use of non-parametric tests for the statistical analysis. The intergroup comparison was performed using Mann-Whitney test. The statistical power was calculated using the GraphPad StatMate tool. All the results were considered statistically significant at the significance level of 5%.

## Supplementary information

Dataset 1

Dataset 2
